# Local adaptation in mainland anole lizards: Integrating population history and genome–environment associations

**DOI:** 10.1002/ece3.4650

**Published:** 2018-11-06

**Authors:** Ivan Prates, Anna Penna, Miguel Trefaut Rodrigues, Ana Carolina Carnaval

**Affiliations:** ^1^ Department of Vertebrate Zoology National Museum of Natural History, Smithsonian Institution Washington District of Columbia; ^2^ Department of Biology, City College of New York and Graduate Center City University of New York New York New York; ^3^ Department of Anthropology University of Texas at San Antonio San Antonio Texas; ^4^ Departamento de Zoologia, Instituto de Biociências Universidade de São Paulo São Paulo Brazil

**Keywords:** Amazonia, *Anolis*, Atlantic Forest, gene flow, phylogeography, population genomics

## Abstract

Environmental gradients constrain physiological performance and thus species’ ranges, suggesting that species occurrence in diverse environments may be associated with local adaptation. Genome–environment association analyses (GEAA) have become central for studies of local adaptation, yet they are sensitive to the spatial orientation of historical range expansions relative to landscape gradients. To test whether potentially adaptive genotypes occur in varied climates in wide‐ranged species, we implemented GEAA on the basis of genomewide data from the anole lizards *Anolis ortonii* and *Anolis punctatus*, which expanded from Amazonia, presently dominated by warm and wet settings, into the cooler and less rainy Atlantic Forest. To examine whether local adaptation has been constrained by population structure and history, we estimated effective population sizes, divergence times, and gene flow under a coalescent framework. In both species, divergence between Amazonian and Atlantic Forest populations dates back to the mid‐Pleistocene, with subsequent gene flow. We recovered eleven candidate genes involved with metabolism, immunity, development, and cell signaling in *A. punctatus* and found no loci whose frequency is associated with environmental gradients in *A. ortonii*. Distinct signatures of adaptation between these species are not associated with historical constraints or distinct climatic space occupancies. Similar patterns of spatial structure between selected and neutral SNPs along the climatic gradient, as supported by patterns of genetic clustering in *A. punctatus*, may have led to conservative GEAA performance. This study illustrates how tests of local adaptation can benefit from knowledge about species histories to support hypothesis formulation, sampling design, and landscape gradient characterization.

## 1 INTRODUCTION

Environmental variation across geographic space limits species’ ranges, therefore affecting ecological processes and large‐scale biogeographic patterns (Ghalambor, Huey, Martin, Tewksbury, & Wang, 2006). Because physiological tolerances to abiotic conditions constrain organismal fitness (Navas, 2002), it has been suggested that the occurrence of any given species across various environments may be associated with local adaptation (Bridle & Vines, 2007; Hohenlohe et al., 2010; Huey, Gilchrist, Carlson, Berrigan, & Serra, 2000). Yet, experimental studies indicate that physiological function can be highly conserved within species over wide environmental gradients (e.g., Crowley, 1985; Hertz, Huey, & Nevo, 1983; John‐Alder, Barnhart, & Bennett, 1989; Prates & Navas, 2009, Prates, Angilleta, Wilson, Niehaus, & Navas, 2013; Van Damme, Bauwens, Castilla, & Verheyen, 1989), and this lack of phenotypic differentiation has been associated with the homogenizing effects of population gene flow (Bridle & Vines, 2007; Lenormand, 2002). It is therefore unclear to which extent the occurrence of species in distinct environments is linked to local adaptation and associated genetic differentiation. Uncovering how environmental gradients contribute to the genetic makeup of organisms, while documenting how population structure and history constrain differentiation, can shed light on the role of local adaptation in the establishment of species in diverse habitats.

To examine local adaptation in heterogeneous landscapes, analyses of genome–environment associations (GEAA) have become a central tool (Rellstab, Gugerli, Eckert, Hancock, & Holderegger, 2015). GEAA studies test for correlations between allele frequencies at multiple loci and environmental predictors across a species' range (Frichot, Schoville, Bouchard, & François, 2013) and are able to account for neutral patterns of population genetic structure that can mimic signatures of selection (Rellstab et al., 2015). However, these analyses are sensitive to the spatial orientation of historical range expansions relative to that of the environmental gradients: A simulation study found that GEAA studies perform best (have lower false discovery rates) when the axes of historical expansion and ecological variation are aligned in space (Frichot, Schoville, Villemereuil, Gaggiotti, & François, 2015). This finding has important implications for empirical studies. First, it suggests that knowledge about the history of occupation of the landscape is crucial to analyses of adaptive genomic differentiation on the basis of GEAA. Second, it implies that studies of local adaptation will benefit from targeting species whose biogeographic trajectories have led to a spatial and ecological arrangement that maximizes GEAA performance.

A system that fits these GEAA requirements well is that of organisms that have undergone range expansions in South American forests. Several species colonized the Atlantic Forest from Amazonia, subsequently expanding southward toward subtropical regions (Batalha‐Filho, Fjeldså, Fabre, & Miyaki, 2013; Costa, 2003; Gehara et al., 2014; Prates, Rivera, Rodrigues, & Carnaval, 2016; Prates, Xue, et al., 2016). It has been shown that Amazonia and the Atlantic Forest experience largely distinct climates (Ledo & Colli, 2017; Sobral‐Souza, Lima‐Ribeiro, & Solferini, 2015). Moreover, pronounced climatic differences distinguish the northern from the southern Atlantic Forest (Carnaval et al., 2014). Species whose ranges now encompass these regions span a climatic gradient from warm and wet conditions in Amazonia to cooler and less rainy settings in the Atlantic Forest. Therefore, these organisms represent an appropriate system to examine signatures of local adaptation associated with species occurrence along environmental gradients on the basis of GEAA. However, the role of genotypes that confer adaptation in shaping the distribution of tropical species remains underexplored (Damasceno, Strangas, Carnaval, Rodrigues, & Moritz, 2014; Moritz, Patton, Schneider, & Smith, 2000; Teixeira, 2016).

This investigation asks whether genetic differentiation identified as potentially adaptive is associated with climatic differentiation along the ranges of the lizards *Anolis ortonii* and *Anolis punctatus*. While these two species belong to anole clades that diverged around 50 mya (Prates et al.., 2017; Prates, Rodrigues, Melo‐Sampaio, & Carnaval, 2015), it has been shown that they underwent similar histories of colonization of the Atlantic Forest from Amazonia (Prates, Rivera, et al., 2016; Prates, Xue, et al., 2016). These lizards are well suited for studies of adaptation. Natural history data indicate that South American forest anoles exhibit low body temperatures when active, and perform little to no behavioral thermoregulation (Vitt, Avila‐Pires, Espósito, Sartorius, & Zani, 2003; Vitt, Avila‐Pires, Zani, Sartorius, & Espósito, 2003; Vitt, Cristina, Avila‐Pires, Zani, & Espósito, 2002; Vitt, Sartorius, Avila‐Pires, & Espósito, 2001)—which points to a role of genetically determined physiological traits in shaping ecological tolerances (e.g., Navas, 2002).

To explore GEAA in this system, we first infer population structure and history within each anole species, estimating levels of population gene flow—a force that could oppose local adaptation. Then, we test whether species establishment in distinct climates is associated with potentially adaptive genetic differentiation between populations. To characterize the climatic gradients presently occupied by *A. ortonii* and *A. punctatus*, we also estimate climatic space occupancy over their ranges.

## 2 MATERIAL AND METHODS

### 2.1 Molecular sampling

We sampled 46 individuals of *Anolis punctatus* and 23 of *Anolis ortonii* (specimen and locality information in Supporting Information Table S1; Supporting Information deposited in the Dryad Digital Repository: https://doi.org/10.5061/dryad.1bj51s9), encompassing a substantial portion of their distributions (Ribeiro‐Júnior, 2015). Reduced representation sequence data were generated as described by Prates, Xue, et al. (2016). Briefly, genomic DNA was extracted from liver fragments preserved in 100% ethanol through a high‐salt extraction protocol following proteinase and RNAase treatment. After examining DNA fragment size using agarose gels, DNA concentration was measured in a Qubit 2 fluorometer (Invitrogen, Waltham) and diluted to ensure a final concentration of 30–100 ng DNA/µl in a total volume of 30 µl (in TE buffer). A restriction site‐associated DNA library was generated through a genotype‐by‐sequencing protocol (GBS; Elshire et al., 2011) at the Institute of Biotechnology at Cornell University. Genomic DNA was digested with the EcoT22I restriction enzyme, and the resulting fragments were tagged with individual barcodes, PCR‐amplified, multiplexed, and sequenced on a single lane on an Illumina HiSeq 2500 platform (rapid run mode). The number of single‐end reads per individual ranged from around 500,000 to six million, with a read length of 100 base pairs. De‐multiplexed raw sequence data were deposited in the Sequence Read Archive (accession PRJNA492310).

We used Ipyrad v. 0.7.23 (Eaton, 2014; available at https://ipyrad.readthedocs.io) to de‐multiplex and assign reads to individuals based on sequence barcodes (allowing no mismatches from individual barcodes), perform de novo read assembly (clustering similarity threshold > 0.9), align reads into loci, and call single‐nucleotide polymorphisms (SNPs). To filter out poor‐quality reads and reduce base‐calling error, we implemented a minimum Phred quality score (=33), minimum sequence coverage (=10×), minimum read length (=70 bp), maximum proportion of heterozygous sites per locus (=0.25), and maximum number of heterozygous individuals per locus (=5) while ensuring that variable sites had no more than two alleles. Following the de‐multiplexing step in Ipyrad, read quality and length were assessed for each sample using FastQC (available at https://www.bioinformatics.babraham.ac.uk/projects/fastqc/). For phylogenetic and genetic clustering analyses, a single SNP was randomly extracted per locus to reduce linkage disequilibrium across sites and to maximize sampling of independent SNP histories. Previous to GEAA and genetic clustering analyses, we used VCFtools v. 0.1.13 (Danecek et al., 2011) to filter out SNPs whose minor allele frequency (MAF) was lower than 0.1 to prevent the inclusion of SNPs that may correspond to sequencing errors (Ahrens et al., 2018).

### 2.2 Inferring population genetic structure

To approximate the number of populations in *A. ortonii* and *A. punctatus*, we estimated the number of genetic clusters (*k*) within each species separately using sNMF v. 1.2 (*Sparse Nonnegative Matrix Factorization*; Frichot, Mathieu, Trouillon, Bouchard, & François, 2014). We tested *k* = 1–10, with 100 replicates for each *k*. The run with the lowest entropy value, estimated by masking 5% of the samples, was considered to identify the best *k* (Frichot et al., 2014). To examine the robustness of sNMF to the regularization parameter (*a*), we ran preliminary analyses with *a* = 1, 10, 100, and 1,000; the best entropy scores were obtained with *a* = 100. Up to 10% missing data were allowed (i.e., each DNA site was present in at least 90% of sampled individuals). The resulting datasets were composed of 2,580 unlinked SNPs for *A. ortonii* and 1,807 for *A. punctatus*.

To further characterize population genetic clustering, we performed a principal component analysis (PCA) based on the SNP data using the *pca* function of the R package LEA v. 2 (*Landscape and Ecological Association*; Frichot & François, 2015).

### 2.3 Inferring population trees

To obtain a population tree for the estimation of gene flow under a historical demographic framework (see below), we inferred phylogenetic relationships between the genetic clusters (thereafter populations) identified by sNMF. Phylogenetic analyses were performed for *A. punctatus* only, for which three populations were recovered; in the case of *A. ortonii*, where only two populations were found based on the genetic data (see Section 3), they were treated as sister to each other in downstream analyses.

To inform historical demographic parameter estimation (see below), a population tree was inferred for *A. punctatus* using SNAPP (*SNP and AFLP Package for Phylogenetic Analysis*; Bryant, Bouckaert, Felsenstein, Rosenberg, & RoyChoudhury, 2012) as implemented in BEAST v. 2.4.8 (*Bayesian Evolutionary Analysis by Sampling Trees*; Bouckaert et al., 2014). A gamma‐distributed prior (given by parameters shape, *α*, and scale, *ρ*) was applied for the *θ* parameter (= 4*Neμ*, where Ne corresponds to the effective population size and *μ* to the per‐generation mutation rate), using *α* = 1 and *ρ* = 0.05 (with mean = 0.05). A gamma prior with *α* = 2 and *ρ* = 100 (with mean = 200) was set to the rate of the Yule diversification process (**λ**) given an expected tree height (*τ* = *Tμ*, where *T* corresponds to the total tree time in number of generations) of ~0.004 substitutions per site based on recent phylogeographic study that included *A. punctatus* (Prates, Rivera, et al., 2016). To help define **λ** prior ranges, we used the python script *Pyule* (available at https://github.com/joaks1/pyule). Default settings were implemented for the mutation parameters. Tree rooting followed Prates, Xue, et al. (2016). To help define prior distributions implemented in SNAPP and G‐PhoCS (see below), we used the *qgamma*,* dgamma*, and *plot* functions in R v. 3.3.3 (R Core Team, 2018).

We performed two independent SNAPP runs of five million generations each, sampling every 1,000 steps. After ensuring convergence between runs and stationarity of model parameters in Tracer v. 1.6 (Drummond, Suchard, Xie, & Rambaut, 2012) and discarding 10% of generations as burn‐in, we visualized posterior tree densities in DensiTree v. 2.4.8 (Bouckaert et al., 2014). Due to computational constraints, population trees were inferred based on five individuals from each of the populations inferred by sNMF, while allowing no missing data. Selected individuals were sampled in sites with no evidence of admixture as inferred by sNMF. The resulting dataset was composed of 2,927 unlinked SNPs across 15 samples of *A. punctatus*.

### 2.4 Estimating historical demographic parameters

To estimate historical demographic parameters such as gene flow, we used G‐PhoCS v. 1.3 (*Generalized Phylogenetic Coalescent Sampler*; Gronau, Hubisz, Gulko, Danko, & Siepel, 2011), which uses the entire nucleotide sequences of loci (as opposed to SNPs only). Populations of *A. ortonii* and *A. punctatus* were defined as the populations inferred through sNMF; historical relationships between populations were defined based on SNAPP results for *A. punctatus*, while the two populations of *A. ortonii* were treated as sister to one another. To assess historical population gene flow, we included bidirectional migration bands for every population pair (i.e., one migration band in each direction).

**Figure 1:**
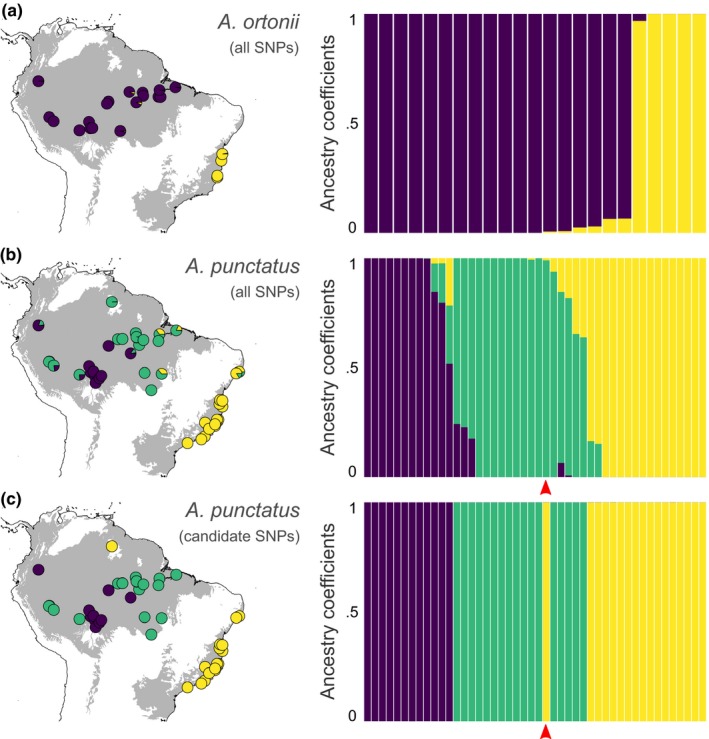
Genetic clustering based on all SNPs from *Anolis ortonii* (a) as well as all SNPs (b) and candidate SNPs only (c) from *Anolis punctatus*. Proportions in pie charts on maps correspond to ancestry coefficients estimated by genetic clustering analyses. Gray areas on map indicate South American rainforests. Red arrows indicate *A. punctatus* sample MTR 20798 from Pacaraima on the Brazil‐Venezuela border in the Guiana Shield region, a locality that is climatically similar to Atlantic Forest sites (see Figure 3); this sample is genetically more similar to eastern Amazonian samples based in the entire SNP dataset, yet more similar to Atlantic Forest samples based on the candidate SNPs only

In G‐PhoCS analyses, we applied gamma distributions (given by parameters shape, *α*, and rate, *β*) for the *θ* and *τ* priors, setting *α* = 1 and *β* = 20 (with mean = 0.05). The gamma prior for each migration band, *m* (=*M*/*μ*, where *M* corresponds to the proportion of individuals in one population that arrived from another population in each generation, and *μ* is the per‐generation mutation rate), was set with *α* = 1 and *β* = 2 × 10^−7^ (with mean = 5,000,000, which corresponds to 1/1,000 of the individuals in one population having arrived from another population in each generation). To convert mutation rate‐scaled parameter estimates to absolute effective population sizes and divergence times, we assumed an average substitution rate of 2.4 × 10^−9^ substitutions per site per year, as estimated by a study that included South American anole species (Prates, Rivera, et al., 2016). To convert estimated coalescent times from the number of generations to years, we assumed a generation time of one year in anoles (Muñoz et al., 2013; Tollis, Ausubel, Ghimire, & Boissinot, 2012).

We performed G‐PhoCS analyses for each species separately implementing a Markov Chain Monte Carlo of 400,000 generations and sampling every 100 generations. The stationarity of each run and the posterior distribution of model parameters were assessed as described for SNAPP analyses. Due to computational constraints, G‐PhoCS analyses used 2,000 randomly selected loci while allowing for no more than 10% missing data.

### 2.5 Choice of environmental variables

As environmental predictors in GEAA, we used bioclimatic variables from the WorldClim database (Hijmans, Cameron, Parra, Jones, & Jarvis, 2005) that describe spatial patterns of temperature and precipitation variation. We limited our analysis to environmental variables that are easily interpretable and that showed clear geographic variation across the distribution of our study anoles: annual mean temperature, maximum temperature of the warmest month, mean temperature of the warmest quarter, mean temperature of the coldest quarter, annual precipitation, precipitation of the wettest month, and precipitation of the wettest quarter. Values were extracted from the collection site of each lizard sample using QGIS v. 2.18.15 (available at https://github.com/qgis/QGIS). Because temperature and precipitation variables were often correlated (pairwise Pearson correlation coefficients >0.7), we followed best practices for GEAA and implemented a PCA on all variables, using the first principal component as a predictor in GEAA (Frichot et al., 2013). PCA was applied using the *prcomp* function in R.

To visualize and compare environmental space occupancy across the range of *A. ortonii* and *A. punctatus*, we used plots of the climatic PC1 against latitude based on sites sampled for genetic data. To better characterize the climatic spaces occupied by these two species, we also compiled a broader dataset that includes georeferenced museum specimens, with a total of 89 and 144 unique records for *A. ortonii* and *A. punctatus*, respectively (Supporting Information Table S2).

### 2.6 Performing genome–environment association analyses

To test for associations between environmental predictors and allele frequencies, we used LFMM (*Latent Factor Mixed Linear Models*; Frichot et al., 2013). LFMM jointly models the effects of environmental variables and neutral genetic structure on allele frequencies, incorporating population structure in the form of unobserved (latent) factors. LFMM has low rates of false positives and is robust to the effects of isolation‐by‐distance and a variety of sampling designs and underlying demographic histories (Rellstab et al., 2015). To define the number of latent factors used in the LFMM analyses, we explored values around the best number of genetic clusters (*k*) inferred by sNMF, as recommended by Frichot et al. (2013). Specifically, we performed preliminary runs incorporating one to six latent factors. Two and five latent factors provided better control of the effects of neutral genetic structure in *A. ortonii* and *A. punctatus*, respectively (see below), and were used in final analyses.

To ensure that false discoveries (i.e., spurious associations between allele frequencies and environmental predictors) were adequately controlled for, we corrected association *p*‐values based on empirical genomic inflation factors (*λ*). These factors are used to modify the baseline null hypothesis in GEAA with the goal of limiting inflation related to population structure and other confounding factors (François, Martins, Caye, & Schoville, 2016). To select *λ*, we inspected histograms of corrected *p*‐values (Supporting Information Figure S1) to ensure that they exhibited a uniform distribution over the interval [0,1] (as expected under the null hypothesis of selective neutrality for most loci) with a peak close to 0 (which corresponds to potentially selected loci; see François et al., 2016). Specifically, we implemented *λ* = 1.5 and 0.45 for *A. ortonii* and *A. punctatus*, respectively. A list of candidate SNPs was generated using the Benjamini–Hochberg algorithm (Benjamini & Hochberg, 1995) by assuming a maximum false discovery rate of 10^−5^.

LFMM was implemented using the *lfmm* function of the LEA R package, with 10 independent runs of 50,000 iterations each and 25,000 iterations as burn‐in. A maximum of 10% missing data was allowed. To reduce the potential effects of missing sites in GEAA, we performed haplotype imputation based on the ancestry coefficients estimated by SNMF, using the *impute* function of the LEA package (as recommended by LFMM developers). Because LFMM makes no assumptions about linkage among SNPs (Dr. Olivier François, personal communication), all SNPs were included in GEAA analyses. After the filtering steps, the genetic dataset was composed of 3,412 SNPs in 2,580 loci for *A. ortonii*, and 3,249 SNPs in 1,920 loci for *A. punctatus*.

R and Unix shell scripts used in all analyses are available online through GitHub (https://github.com/ivanprates/2018_Anolis_EcolEvol).

### 2.7 Candidate gene identity and function

LFMM analyses recovered SNPs whose frequencies are linked to environmental gradients (see Section 3.4). To examine whether the candidate loci harboring these SNPs correspond to genes, we used the BLASTN algorithm (McGinnis & Madden, 2004) as implemented in Ensembl v. 91 (available at https://useast.ensembl.org/Multi/Tools/Blast) to map each sequence against the reference genome of *Anolis carolinensis* (assembly AnoCar2.0; Alföldi et al., 2011). For blasting, we used a maximum *e*‐value of 10^−5^ while masking low complexity (e.g., repetitive) regions. In the case of mapped loci that correspond to protein‐coding regions, we then examined gene identity and function as summarized by gene ontology terms based on the UniProt database (available at https://www.uniprot.org/uniprot/).

## 3 RESULTS

### 3.1 Population genetic structure

Clustering analyses using SNMF inferred higher levels of population structure in *Anolis punctatus* than in *Anolis ortonii*, with three and two populations recovered in each species, respectively (Figure 1a,b). However, the two species show similarities in the patterns of population structure across space. For instance, Atlantic Forest samples belong to a distinct population relative to Amazonian samples in both species, which suggests genetic differentiation across forest blocks. On the other hand, only one population of *A. ortonii* was recovered in Amazonia, whereas two populations of *A. punctatus* were inferred in that region. In *A. punctatus*, one population is widely distributed in central and eastern Amazonia, also including samples from western Amazonia near the Brazil‐Peru border, whereas another one extends from southwestern Amazonia (around the Madeira River) northwards (Figure 1b).

Genetic clustering analyses recovered admixture (as indicated by individual ancestry coefficients) between Atlantic Forest and Amazonian populations in both *A. ortonii* and *A. punctatus* (Figure 1a,b), with higher levels of admixture in the latter. In the case of *A. punctatus*, admixed individuals were sampled in the northern extreme of the Atlantic Forest, close to Amazonia's eastern edge, and in western Amazonia (Figure 1b).

### 3.2 Population history

**Figure 2:**
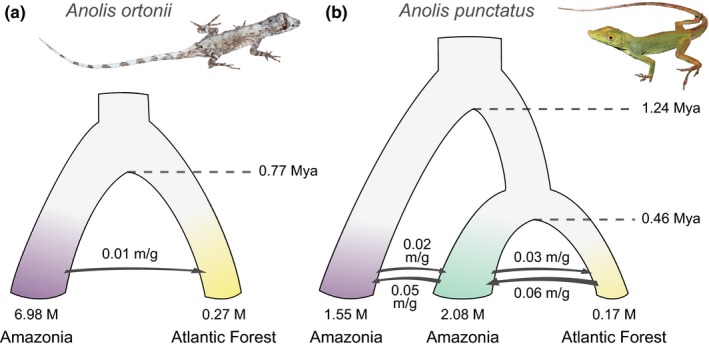
Population history (from SNAPP) and historical demographic parameters (from G‐PhoCS) inferred for *Anolis ortonii* (a) and *A. punctatus* (b). Parameters are the time of coalescence between populations (in millions of years, Mya), effective population sizes (in millions of individuals, M), and migration rates (in migrants per generation, m/g). Colors of terminals correspond to genetic clusters in Figure 1

A scenario of early separation between Amazonian and Atlantic Forest populations in *A. ortonii* differs from the pattern seen in *A. punctatus*. In *A. punctatus*, phylogenetic analyses using SNAPP found that the Atlantic Forest population is nested among Amazonian samples (Figure 2). This Atlantic population is the sister of the population that occurs in eastern Amazonia; the clade formed by them is sister to that population that extends from southwestern Amazonia (around the Madeira River) northwards.

G‐PhoCS analyses inferred large effective population sizes in all regions for both *A. ortonii* and *A. punctatus*, estimated as hundreds of thousands to millions of individuals (Figure 2). Coalescent times between the Atlantic Forest population and the most closely related Amazonian lineage date back to the mid‐Pleistocene in both anole species. We found a signal of historical gene flow between populations that occur in Amazonia and the Atlantic Forest in both species, with levels of population gene flow being overall higher in *A. punctatus* than in *A. ortonii* (Figure 2).

### 3.3 Climatic space occupancy

**Figure 3:**
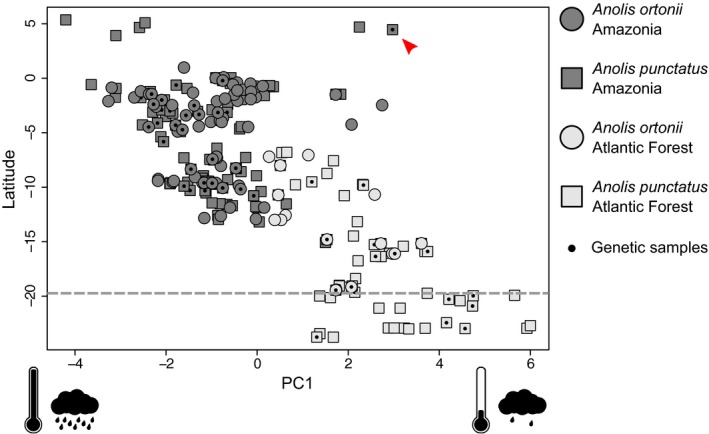
Environmental space occupancy along latitude based on climatic PC1 for *Anolis ortonii* and *A. punctatus*. Samples used in genetic analyses are indicated with a black dot. Higher PC scores correspond to drier and colder sites. Dashed line indicates the approximate region of pronounced north–south climatic turnover in the Atlantic Forest as identified by Carnaval et al. (2014). Red arrow indicates *A. punctatus* sample MTR 20798 from Pacaraima, a mid‐elevation site (820 m above sea level) in the Guiana Shield region that overlaps climatically with Atlantic Forest sites (horizontal axis)

The first component of the PCA based on temperature and precipitation variables (PC1), used as a predictor variable in GEAA, explains >70% of the total climatic variation across sampled sites in both species. Positive scores in climate PC1 correspond to lower temperature and precipitation values (Supporting Information Table S3). Plots of climatic space occupancy along a latitudinal gradient based on PC1 suggest that both species experience similar climates over their range in Amazonia (Figure 3). However, while *A. punctatus* occupies cooler and less rainy environments in the southern end of its distribution in the Atlantic Forest, *A. ortonii* is restricted to warmer and more humid sites in the northern Atlantic Forest, which is climatically more similar to Amazonia. This pattern holds when considering both sites sampled for genetic data and a broader dataset that also includes hundreds of occurrence records from museum specimens (Figure 3).

### 3.4 Genome–environment association analyses

GEAA controlling for neutral genetic structure using LFMM found allele frequencies of 86 SNPs in 39 loci to be significantly associated with climatic gradients in *A. punctatus*. Among these loci, 30 blasted against regions of the annotated reference genome of *A. carolinensis*. Eleven loci uniquely mapped to known protein‐coding genes (Table 1); two candidate loci mapped non‐specifically to more than four genes; and the remaining loci blasted against non‐coding regions, which may correspond to regions that regulate gene expression (e.g., enhancers or transcription factor recognition sites) or that are physically linked to genes that underwent selection.

**Table 1:** Candidate loci identified by genome–environment association analyses using LFMM for *Anolis punctatus*

GBS locus	*Anolis carolinensis* chromosome (or scaffold)	Position (bp)	Overlapping gene	Match length (bp)	*E*‐value	Identity (%)
48380	LGa	3,044,340–3,044,411	CXADR‐like membrane protein gene (CLMP)	72	1E−20	93.06
52588	GL343225	1,347,775–1,347,853	PHD finger protein 2 (PHF2)	79	1E−22	92.41
57548	4	91,951,599–91,951,684	Dihydropyrimidine dehydrogenase gene (DPYD)	86	1E−19	89.53
58069	5	140,581,178–140,581,220	*N*‐deacetylase and *N*‐sulfotransferase 3 gene (NDST3)	43	9E−06	90.70
65078	1	111,909,958–111,910,047	Teneurin transmembrane protein 2 (TENM2)	90	1E−38	97.78
68654	3	58,901,943–58,902,027	Inositol polyphosphate−5‐phosphatase A (INPP5A)	85	2E−16	88.24
72522	GL343384	248,338–248,397	Lymphoid enhancer binding factor 1 (LEF1)	55	7E−06	87.27
74703	GL343290	1,535,974–1,721,555	Collagen type V alpha 2 chain (COL5A2)	80	1E−13	87.50
74803	1	197,640,485–197,640,538	Malic enzyme 1 gene (ME1)	54	9E−15	94.44
75555	2	120,299,259–120,299,348	Dedicator of cytokinesis 2 gene (DOCK2)	90	1E−19	88.89
77416	1	101,850,853–101,850,977	Rho GTPase activating protein 15 (ARHGAP15)	114	2E−50	97.37

Note

Only genes that successfully blasted against known protein‐coding regions of the genome of *A. carolinensis* are shown.

In the case of *A. ortonii*, GEAA found no evidence of genomic differentiation associated with environmental gradients. In this species, no SNPs were linked with temperature and precipitation variation across space, even when relaxing the false discovery rate from 10^−5^ to 10^−3^.

Inspection of read quality using FastQC indicated similar high‐quality reads for individuals of both species (not shown), suggesting that differences in DNA quality do not account for distinct GEAA results between *A. punctatus* and *A. ortonii*.

### 3.5 Patterns of candidate allele sharing among populations

In *A. punctatus*, genetic clustering analyses based solely on the candidate SNPs suggest no allele sharing between populations in distinct regions (Figure 1c), partially mirroring sNMF results based on the entire SNP dataset (Figure 1b). Interestingly, one sample from the Guiana Shield region (MTR 20798 from Pacaraima, state of Roraima in Brazil) clusters with Atlantic Forest samples in a clustering analysis based on the candidate SNPs only (Figure 1c). By contrast, this sample clusters with eastern Amazonian samples in an analysis based on the entire SNP dataset (Figure 1b). A genetic PCA based on these two SNP datasets shows the same pattern (Supporting Information Figure S2).

## 4 DISCUSSION

Based on genomewide data of *A. ortonii* and *A. punctatus*, two widespread South American anole lizard species, we infer distinct genetic populations in Amazonia and the Atlantic Forest, suggesting that separation of these forests following a period of contact has favored genetic divergence (Figure 1). In both species, historical demographic analyses indicate large effective population sizes, mid‐Pleistocene colonizations, and post‐divergence population gene flow (Figure 2). Lastly, we find evidence of associations between environmental gradients and allele frequency patterns in *A. punctatus*, but not in *A. ortonii* (Table 1), which is consistent with local adaptation in *A. punctatus* only. Analyses of environmental space occupancy suggest that the two species experience largely similar climates in Amazonia and the northern Atlantic Forest, yet *A. punctatus* occupies cooler and less humid localities that are not occupied by *A. ortonii* in the southern Atlantic Forest (Figure 3).

### 4.1 Population history of South American forest anoles

Historical relationships and coalescent times between Amazonian and Atlantic Forest populations of both *A. ortonii* and *A. punctatus* support the hypothesis that a forest corridor connected these two major forest blocks in the mid‐Pleistocene. These findings corroborate the results of previous analyses based on much smaller genetic datasets for these species (Prates et al., 2015; Prates, Rivera, et al., 2016). Moreover, they agree with paleontological, geochemical, and palynological records supporting a history of pulses of increased precipitation in South America during the Quaternary. Wetter climates have been implicated in periodic forest expansions in present‐day northeastern Brazil, leading to connections and species exchange between Amazonia and the Atlantic Forest (e.g., Cheng et al., 2013; de Oliveira, Barreto, & Suguio, 1999; de Vivo & Carmignotto, 2004).

Recurrent forest corridors may have led to periodic re‐establishment of gene flow following the colonization of new regions. A previous test of this idea, based on a multi‐locus dataset from *A. ortonii* and *A. punctatus*, was inconclusive, presumably due to limited signal in the genetic data available (Prates, Rivera, et al., 2016). Here, on the basis thousands of restriction site‐associated markers, we found evidence of historical gene flow across major forest blocks. The genetic clustering analyses found admixed individuals in eastern Amazonia and northern sites in the Atlantic Forest (Figure 1a,b). Moreover, the estimation of historical demographic parameters under a coalescent framework recovered population gene flow across forests in both anole species (Figure 2). These results are consistent with the idea of recurrent forest expansions that favored post‐divergence migration across regions. It is currently unclear, however, whether this signature of historical gene flow resulted from one or multiple forest connections over time.

Genetic clustering analyses of *A. ortonii* and *A. punctatus* found that Atlantic Forest samples compose a distinct genetic group from Amazonian samples in both species, pointing to an important role of forest separation in promoting population genetic divergence. The idea of expansion of semi‐arid habitats in northeastern Brazil during the Quaternary, causing fragmentation of forest habitats, is supported by genetic studies of species from these dry regions (Gehara et al., 2017; Thomé et al., 2016). Former expansions of open and dry habitats may have favored speciation and further diversification of forest organisms, as supported by a pattern of sister relationships between species or clades that occur in Amazonia and the Atlantic Forest as seen in birds, snakes, and mammals (e.g., Costa, 2003; Batalha‐Filho et al., 2013; Dal‐Vechio, Prates, Grazziotin, Zaher, & Rodrigues, 2018).

### 4.2 Candidate genes point to ecologically relevant physiological processes

GEAA results provide insights about physiological processes that may have experienced selection in response to climatic regimes in *A. punctatus*. The candidate genes identified play important roles in energy metabolism, immunity, development, and cell signaling. For instance, the *malic enzyme 1* gene (ME1) encodes an enzyme that links the glycolytic and citric acid pathways (Jiang, Du, Mancuso, Wellen, & Yang, 2013) while generating NADPH necessary for fatty acid biosynthesis (Bukato, Kochan, & Świerczyński, 1995). The *collagen type V alpha 2 chain* gene (COL5A2) encodes a structural component of the extracellular matrix that is involved with skeletal, skin, and eye development through collagen fibril organization (Andrikopoulos, Liu, Keene, Jaenisch, & Ramirez, 1995). The product of the *dedicator of cytokinesis 2* gene (DOCK2) is crucial in immune response by mediating the differentiation of T cells and lymphocyte migration to sites of infection (Fukui et al., 2001; Kunisaki et al., 2006). Finally, the *coxsackie‐ and adenovirus receptor‐like membrane protein* gene (CLMP) encodes a transmembrane component of cell‐to‐cell junctional complexes that is essential for intestine development (Raschperger, Engstrom, Pettersson, & Fuxe, 2003). By pointing to ecologically relevant physiological processes, candidate genes recovered by GEAA can guide eco‐physiological investigations of South American lizards. Future experimental studies that compare physiological tolerances between individuals from distinct habitats have the potential to reveal links between patterns of genetic variation, organismal function, and species distributions (Navas, 2002).

Similar to this study, other investigations found differences in the frequency of alleles that underlie ecologically relevant physiological processes between populations that inhabit contrasting habitats (e.g., Yoder et al., 2014; Benestan et al., 2016; Dennenmoser, Vamosi, Nolte, & Rogers, 2017; Guo, Lu, Liao, & Merilä, 2016). A few of these studies involved anole lizards. For instance, in the North American *A. carolinensis*, colonization of colder temperate regions from a tropical Caribbean source has been linked to adaptive shifts in several genes, including loci that are involved with development, energy metabolism, and immune response (Campbell‐Staton, Edwards, & Losos, 2016). Additionally, evidence of thermal adaptation along an altitudinal gradient has been found in *Anolis cybotes* from Hispaniola, involving genes that play a role in bone formation and development, lipid metabolism, and regulation of blood pressure (Rodríguez et al., 2017). Similar to the case of *A. punctatus*, these examples support that adaptation to cooler climates has played an essential role in range expansions across anole taxa, including mainland and Caribbean forms that span altitudinal and latitudinal gradients. It is currently unclear, however, whether potentially beneficial polymorphisms preceded the colonization of new geographic regions by these species.

### 4.3 Parallels between Guiana Shield and Atlantic Forest anoles

An individual of *A. punctatus* (MTR 20798) sampled in a mid‐elevation site (820 m above sea level) in the Guiana Shield, on the Brazil‐Venezuela border, grouped with Atlantic Forest samples in a clustering analysis based solely on candidate SNPs (Figure 1c, Supporting Information Figure S2b). By contrast, this sample clustered with eastern Amazonian samples (which are geographically closer) in an analysis based on the entire SNP dataset (Figure 1b, Supporting Information Figure S2a). Interestingly, the site where this individual was collected from is climatically more similar to Atlantic Forest than to lowland Amazonian sites (Figure 3). These results may suggest that *A. punctatus* in the Guiana highlands and in the Atlantic Forest have independently acquired alleles that are beneficial under cooler or less rainy conditions, because this study (Figure 1b, Supporting Information Figure S2a) and previous analyses of historical relationships based on individual samples of *A. punctatus* (Prates, Rivera, et al., 2016; Prates, Xue, et al., 2016) found that Guiana Shield samples are not closely related to Atlantic Forest ones. Alternatively, this pattern may reflect differences in how neutral and beneficial alleles spread in geographic space, as demonstrated in other animal species that occur in contrasting habitats (Hoekstra, Drumm, & Nachman, 2004). Future analyses of local adaptation in populations that occur in the Guiana highlands will rely on improved sampling for this key region.

### 4.4 Distinct GEAA patterns among species

Population structure and history can constrain local adaptation (Bridle & Vines, 2007; Lenormand, 2002), yet our analyses suggest that these factors do not account for different signatures of adaptation between the two anole species. G‐PhoCS analyses recovered higher gene flow in *A. punctatus* than in *A. ortonii*, as well as older colonization of the Atlantic Forest in *A. ortonii* than in *A. punctatus* (Figure 2). Lower levels of gene flow and earlier colonization might have favored local adaptation in *A. ortonii*; however, GEAA found allele frequency patterns associated with climatic gradients in *A. punctatus* only. As a result, constraints related to population structure and history do not seem sufficient to explain discrepant GEAA results between the two species.

Alternatively, distinct GEAA patterns in *A. ortonii* and *A. punctatus* might stem from differences in the climatic regimes experienced by each species in parts of their range. In the coastal Atlantic Forest, *A. ortonii* is restricted to northern regions that are climatically more similar to Amazonia (Ledo & Colli, 2017; Sobral‐Souza et al., 2015; Figure 3). By contrast, *A. punctatus* extends into colder and less rainy areas in the southern Atlantic Forest (Figure 3), as far as the state of São Paulo in Brazil's southeast. As a result, our finding of several candidate SNPs in *A. punctatus* might be associated with local adaptation to cooler and less rainy conditions in the southern edge of this species’ range, as opposed to a pattern of physiological conservatism in *A. ortonii*, which shows a narrower range in the Atlantic Forest. Different species capacities to adapt to regional climates may hold the key to a pattern of pronounced taxonomic composition turnover around 19–20° of latitude in coastal Brazil (Carnaval et al., 2014). However, genetic clustering (Figure 1c) and PCA analyses (Supporting Information Figure S2b) based on the candidate SNPs flagged by GEAA do not support a pattern of differentiation between northern and southern Atlantic Forest samples of *A. punctatus*.

Lastly, it is possible that contrasting GEAA results in *A. ortonii* and *A. punctatus* have been influenced by sampling differences. Fewer individuals of *A. ortonii* were available for study, reflecting difficulties to sample this less abundant and less conspicuous species in the field. Limited sample sizes can strongly affect the power of GEAA to detect loci whose frequencies are associated with environmental factors. This limitation is particularly important in the case of genetic variants that have weak environmental associations, which can be detected only with large sample sizes (Murcray, Lewinger, Conti, Thomas, & Gauderman, 2011). It is therefore possible that GEAA recovered only those candidate SNPs that are more strongly associated with temperature and precipitation gradients across the range of *A. punctatus* yet did not detect several other potentially beneficial SNPs in this species and also in *A. ortonii*. This situation illustrates the challenges of implementing GEAA on the basis of samples from natural populations, which can be hard to obtain in large numbers for certain organisms. Nevertheless, the suggestion of loci that show strong genome–environment associations in *A. punctatus* encourages further tests on the basis of larger sample sizes.

### 4.5 GEAA in species that have expanded along environmental gradients

Our analyses of local adaptation may have been affected by conservative GEAA performance in species that underwent a history of range expansions along environmental gradients. When the axis of range expansion is parallel to the orientation of ecological gradients, the selected sites are expected to exhibit patterns of spatial structure that are similar to those of the neutral sites. As a result, the test's null hypothesis (which relies on neutral background genetic variation) yields a stricter control of false positives (Frichot et al., 2015), and GEAA is expected to detect only the outlier loci that exhibit strong associations with the environmental predictor. A similar pattern of spatial structure between presumably selected and neutral sites agrees with our observation of concordant population clustering based on both candidate and neutral SNPs in *A. punctatus* (Figure 1b,c; Supporting Information Figure S2). While minimizing spurious genome–environment associations, this situation may also increase the rate of undetected adaptive genotypes. Conservative tests of adaptation in species that have expanded along environments gradients may be intensified in instances of limited spatial and individual sampling, as it may have been the case of *A. ortonii*.

This investigation illustrates how studies of adaptation on the basis of GEAA can benefit from knowledge about the history of landscape occupation by the species under investigation. Data on population structure and history can provide insight about how gene flow and natural selection interact and shape population genetic differentiation. Moreover, information about the direction and routes of colonization of new habitats can support spatial sampling design, help to characterize landscape gradients, and support the formulation of hypotheses about how organisms have responded to environmental variation in space.

## CONFLICT OF INTEREST

None declared.

## AUTHOR CONTRIBUTIONS

I.P., A.P., M.T.R., and A.C.C. designed research; I.P. and A.P. performed research; M.T.R. and A.C.C. contributed new reagents/analytic tools; I.P., A.P., M.T.R., and A.C.C. analyzed data; and I.P., A.P., M.T.R., and A.C.C. wrote the paper.

## DATA ACCESSIBILITY

All sequence data were deposited in the Sequence Read Archive (accession PRJNA492310). Supplementary tables and figures were deposited in the Dryad Digital Repository (https://doi.org/10.5061/dryad.1bj51s9). R and Unix shell scripts used in all analyses are available online through GitHub (https://github.com/ivanprates/2018_Anolis_EcolEvol).

## References

[ece34650-bib-0001] Ahrens, C. W. , Rymer, P. D. , Stow, A. , Bragg, J. , Dillon, S. , Umbers, K. D. , & Dudaniec, R. Y. (2018). The search for loci under selection: Trends, biases and progress. Molecular Ecology, 27(6), 1342–1356. 10.1111/mec.14549 29524276

[ece34650-bib-0002] Alföldi, J. , Di Palma, F. , Grabherr, M. , Williams, C. , Kong, L. , Mauceli, E. , … Ray, D. A. (2011). The genome of the green anole lizard and a comparative analysis with birds and mammals. Nature, 477(7366), 587–591. 21881562 10.1038/nature10390 PMC3184186

[ece34650-bib-0003] Andrikopoulos, K. , Liu, X. , Keene, D. R. , Jaenisch, R. , & Ramirez, F. (1995). Targeted mutation in the col5a2 gene reveals a regulatory role for type V collagen during matrix assembly. Nature Genetics, 9(1), 31–36. 10.1038/ng0195-31 7704020

[ece34650-bib-0004] Batalha‐Filho, H. , Fjeldså, J. , Fabre, P. H. , & Miyaki, C. Y. (2013). Connections between the Atlantic and the Amazonian forest avifaunas represent distinct historical events. Journal of Ornithology, 154(1), 41–50. 10.1007/s10336-012-0866-7

[ece34650-bib-0005] Benestan, L. , Quinn, B. K. , Maaroufi, H. , Laporte, M. , Clark, F. K. , Greenwood, S. J. , … Bernatchez, L. (2016). Seascape genomics provides evidence for thermal adaptation and current‐mediated population structure in American lobster (*Homarus americanus*). Molecular Ecology, 25(20), 5073–5092. 27543860 10.1111/mec.13811

[ece34650-bib-0006] Benjamini, Y. , & Hochberg, Y. (1995). Controlling the false discovery rate: A practical and powerful approach to multiple testing. Journal of the Royal Statistical Society. Series B (Methodological), 57(1), 289–300.

[ece34650-bib-0007] Bouckaert, R. , Heled, J. , Kühnert, D. , Vaughan, T. , Wu, C. H. , Xie, D. , … Drummond, A. J. (2014). BEAST 2: A software platform for Bayesian evolutionary analysis. PLoS Computational Biology, 10(4), e1003537 10.1371/journal.pcbi.1003537 24722319 PMC3985171

[ece34650-bib-0008] Bridle, J. R. , & Vines, T. H. (2007). Limits to evolution at range margins: When and why does adaptation fail? Trends in Ecology & Evolution, 22(3), 140–147. 10.1016/j.tree.2006.11.002 17113679

[ece34650-bib-0009] Bryant, D. , Bouckaert, R. , Felsenstein, J. , Rosenberg, N. A. , & RoyChoudhury, A. (2012). Inferring species trees directly from biallelic genetic markers: Bypassing gene trees in a full coalescent analysis. Molecular Biology and Evolution, 29(8), 1917–1932. 10.1093/molbev/mss086 22422763 PMC3408069

[ece34650-bib-0010] Bukato, G. , Kochan, Z. , & Świerczyński, J. (1995). Purification and properties of cytosolic and mitochondrial malic enzyme isolated from human brain. The International Journal of Biochemistry & Cell Biology, 27(1), 47–54. 10.1016/1357-2725(94)00057-3 7757881

[ece34650-bib-0011] Campbell‐Staton, S. C. , Edwards, S. V. , & Losos, J. B. (2016). Climate‐mediated adaptation after mainland colonization of an ancestrally subtropical island lizard, *Anolis carolinensis* . Journal of Evolutionary Biology, 29(11), 2168–2180. 27384884 10.1111/jeb.12935

[ece34650-bib-0012] Carnaval, A. C. , Waltari, E. , Rodrigues, M. T. , Rosauer, D. , VanDerWal, J. , Damasceno, R. , … Pie, M. R. (2014). Prediction of phylogeographic endemism in an environmentally complex biome. Proceedings of the Royal Society B: Biological Sciences, 281(1792), 20141461 10.1098/rspb.2014.1461 PMC4150330 25122231

[ece34650-bib-0013] Cheng, H. , Sinha, A. , Cruz, F. W. , Wang, X. , Edwards, R. L. , d'Horta, F. M. , … Auler, A. S. (2013). Climate change patterns in Amazonia and biodiversity. Nature Communications, 4, 1411 10.1038/ncomms2415 23361002

[ece34650-bib-0014] Costa, L. P. (2003). The historical bridge between the Amazon and the Atlantic Forest of Brazil: A study of molecular phylogeography with small mammals. Journal of Biogeography, 30(1), 71–86. 10.1046/j.1365-2699.2003.00792.x

[ece34650-bib-0015] Crowley, S. R. (1985). Thermal sensitivity of sprint‐running in the lizard *Sceloporus undulatus*: Support for a conservative view of thermal physiology. Oecologia, 66(2), 219–225. 10.1007/BF00379858 28311593

[ece34650-bib-0016] Dal‐Vechio, F. , Prates, I. , Grazziotin, F. G. , Zaher, H. , & Rodrigues, M. T. (2018). Phylogeography and historical demography of the arboreal pit viper *Bothrops bilineatus* (Serpentes, Crotalinae) reveal multiple connections between Amazonian and Atlantic rain forests. Journal of Biogeography, 45, 2415–2426.

[ece34650-bib-0017] Damasceno, R. , Strangas, M. L. , Carnaval, A. C. , Rodrigues, M. T. , & Moritz, C. (2014). Revisiting the vanishing refuge model of diversification. Frontiers in Genetics, 5, 353 10.3389/fgene.2014.00353 25374581 PMC4205810

[ece34650-bib-0018] Danecek, P. , Auton, A. , Abecasis, G. , Albers, C. A. , Banks, E. , DePristo, M. A. , … McVean, G. (2011). The variant call format and VCFtools. Bioinformatics, 27(15), 2156–2158. 10.1093/bioinformatics/btr330 21653522 PMC3137218

[ece34650-bib-0019] de Oliveira, P. E. , Barreto, A. M. F. , & Suguio, K. (1999). Late Pleistocene/Holocene climatic and vegetational history of the Brazilian caatinga: The fossil dunes of the middle São Francisco River. Palaeogeography, Palaeoclimatology, Palaeoecology, 152(3–4), 319–337. 10.1016/S0031-0182(99)00061-9

[ece34650-bib-0020] de Vivo, M. , & Carmignotto, A. P. (2004). Holocene vegetation change and the mammal faunas of South America and Africa. Journal of Biogeography, 31(6), 943–957. 10.1111/j.1365-2699.2004.01068.x

[ece34650-bib-0021] Dennenmoser, S. , Vamosi, S. M. , Nolte, A. W. , & Rogers, S. M. (2017). Adaptive genomic divergence under high gene flow between freshwater and brackish‐water ecotypes of prickly sculpin (*Cottus asper*) revealed by Pool‐Seq. Molecular Ecology, 26(1), 25–42. 27541083 10.1111/mec.13805

[ece34650-bib-0022] Drummond, A. J. , Suchard, M. A. , Xie, D. , & Rambaut, A. (2012). Bayesian phylogenetics with BEAUti and the BEAST 1.7. Molecular Biology and Evolution, 29(8), 1969–1973. 10.1093/molbev/mss075 22367748 PMC3408070

[ece34650-bib-0023] Eaton, D. A. (2014). PyRAD: Assembly of de novo RADseq loci for phylogenetic analyses. Bioinformatics, 30(13), 1844–1849. 10.1093/bioinformatics/btu121 24603985

[ece34650-bib-0024] Elshire, R. J. , Glaubitz, J. C. , Sun, Q. , Poland, J. A. , Kawamoto, K. , Buckler, E. S. , & Mitchell, S. E. (2011). A robust, simple genotyping‐by‐sequencing (GBS) approach for high diversity species. PLoS ONE, 6(5), e19379 10.1371/journal.pone.0019379 21573248 PMC3087801

[ece34650-bib-0025] François, O. , Martins, H. , Caye, K. , & Schoville, S. D. (2016). Controlling false discoveries in genome scans for selection. Molecular Ecology, 25(2), 454–469. 10.1111/mec.13513 26671840

[ece34650-bib-0026] Frichot, E. , & François, O. (2015). LEA: An R package for landscape and ecological association studies. Methods in Ecology and Evolution, 6(8), 925–929.

[ece34650-bib-0027] Frichot, E. , Mathieu, F. , Trouillon, T. , Bouchard, G. , & François, O. (2014). Fast and efficient estimation of individual ancestry coefficients. Genetics, 196, 973–983. 10.1534/genetics.113.160572 24496008 PMC3982712

[ece34650-bib-0028] Frichot, E. , Schoville, S. D. , Bouchard, G. , & François, O. (2013). Testing for associations between loci and environmental gradients using latent factor mixed models. Molecular Biology and Evolution, 30(7), 1687–1699. 10.1093/molbev/mst063 23543094 PMC3684853

[ece34650-bib-0029] Frichot, E. , Schoville, S. D. , de Villemereuil, P. , Gaggiotti, O. E. , & François, O. (2015). Detecting adaptive evolution based on association with ecological gradients: Orientation matters!. Heredity, 115(1), 22–28. 10.1038/hdy.2015.7 25690180 PMC4815498

[ece34650-bib-0030] Fukui, Y. , Hashimoto, O. , Sanui, T. , Oono, T. , Koga, H. , Abe, M. , … Sasazuki, T. (2001). Haematopoietic cell‐specific CDM family protein DOCK2 is essential for lymphocyte migration. Nature, 412(6849), 826–831. 11518968 10.1038/35090591

[ece34650-bib-0031] Gehara, M. , Crawford, A. J. , Orrico, V. G. , Rodriguez, A. , Loetters, S. , Fouquet, A. , … Urrutia, G. G. (2014). High levels of diversity uncovered in a widespread nominal taxon: Continental phylogeography of the Neotropical tree frog *Dendropsophus minutus* . PLoS ONE, 9(9), e103958 10.1371/journal.pone.0103958 25208078 PMC4160190

[ece34650-bib-0032] Gehara, M. , Garda, A. A. , Werneck, F. P. , Oliveira, E. F. , da Fonseca, E. M. , Camurugi, F. , … Silveira‐Filho, R. (2017). Estimating synchronous demographic changes across populations using hABC and its application for a herpetological community from northeastern Brazil. Molecular Ecology, 26(18), 4756–4771. 10.1111/mec.14239 28734050

[ece34650-bib-0033] Ghalambor, C. K. , Huey, R. B. , Martin, P. R. , Tewksbury, J. J. , & Wang, G. (2006). Are mountain passes higher in the tropics? Janzen's hypothesis revisited. Integrative and Comparative Biology, 46(1), 5–17. 10.1093/icb/icj003 21672718

[ece34650-bib-0034] Gronau, I. , Hubisz, M. J. , Gulko, B. , Danko, C. G. , & Siepel, A. (2011). Bayesian inference of ancient human demography from individual genome sequences. Nature Genetics, 43(10), 1031–1034. 10.1038/ng.937 21926973 PMC3245873

[ece34650-bib-0035] Guo, B. , Lu, D. , Liao, W. B. , & Merilä, J. (2016). Genomewide scan for adaptive differentiation along altitudinal gradient in the Andrew's toad *Bufo andrewsi* . Molecular Ecology, 25(16), 3884–3900. 27289071 10.1111/mec.13722

[ece34650-bib-0036] Hertz, P. E. , Huey, R. B. , & Nevo, E. (1983). Homage to Santa Anita: Thermal sensitivity of sprint speed in agamid lizards. Evolution, 37(5), 1075–1084. 10.1111/j.1558-5646.1983.tb05634.x 28563551

[ece34650-bib-0037] Hijmans, R. J. , Cameron, S. E. , Parra, J. L. , Jones, P. G. , & Jarvis, A. (2005). Very high resolution interpolated climate surfaces for global land areas. International Journal of Climatology, 25(15), 1965–1978. 10.1002/joc.1276

[ece34650-bib-0038] Hoekstra, H. E. , Drumm, K. E. , & Nachman, M. W. (2004). Ecological genetics of adaptive color polymorphism in pocket mice: Geographic variation in selected and neutral genes. Evolution, 58(6), 1329–1341. 10.1111/j.0014-3820.2004.tb01711.x 15266981

[ece34650-bib-0039] Hohenlohe, P. A. , Bassham, S. , Etter, P. D. , Stiffler, N. , Johnson, E. A. , & Cresko, W. A. (2010). Population genomics of parallel adaptation in threespine stickleback using sequenced RAD tags. PLoS Genetics, 6(2), e1000862 10.1371/journal.pgen.1000862 20195501 PMC2829049

[ece34650-bib-0040] Huey, R. B. , Gilchrist, G. W. , Carlson, M. L. , Berrigan, D. , & Serra, L. (2000). Rapid evolution of a geographic cline in size in an introduced fly. Science, 287(5451), 308–309. 10634786 10.1126/science.287.5451.308

[ece34650-bib-0041] Jiang, P. , Du, W. , Mancuso, A. , Wellen, K. E. , & Yang, X. (2013). Reciprocal regulation of p53 and malic enzymes modulates metabolism and senescence. Nature, 493(7434), 689–693. 23334421 10.1038/nature11776 PMC3561500

[ece34650-bib-0042] John‐Alder, H. B. , Barnhart, M. C. , & Bennett, A. F. (1989). Thermal sensitivity of swimming performance and muscle contraction in northern and southern populations of tree frogs (*Hyla crucifer*). Journal of Experimental Biology, 142(1), 357–372.

[ece34650-bib-0044] Kunisaki, Y. , Tanaka, Y. , Sanui, T. , Inayoshi, A. , Noda, M. , Nakayama, T. , … Fukui, Y. (2006). DOCK2 is required in T cell precursors for development of Vα14 NK T cells. The Journal of Immunology, 176(8), 4640–4645. 16585555 10.4049/jimmunol.176.8.4640

[ece34650-bib-0045] Ledo, R. M. D. , & Colli, G. R. (2017). The historical connections between the Amazon and the Atlantic Forest revisited. Journal of Biogeography, 44(11), 2551–2563. 10.1111/jbi.13049

[ece34650-bib-0046] Lenormand, T. (2002). Gene flow and the limits to natural selection. Trends in Ecology & Evolution, 17(4), 183–189. 10.1016/S0169-5347(02)02497-7

[ece34650-bib-0048] McGinnis, S. , & Madden, T. L. (2004). BLAST: At the core of a powerful and diverse set of sequence analysis tools. Nucleic Acids Research, 32(suppl_2), W20–W25. 10.1093/nar/gkh435 15215342 PMC441573

[ece34650-bib-0049] Moritz, C. , Patton, J. L. , Schneider, C. J. , & Smith, T. B. (2000). Diversification of rainforest faunas: An integrated molecular approach. Annual Review of Ecology and Systematics, 31(1), 533–563. 10.1146/annurev.ecolsys.31.1.533

[ece34650-bib-0050] Muñoz, M. M. , Crawford, N. G. , McGreevy, T. J. Jr , Messana, N. J. , Tarvin, R. D. , Revell, L. J. , … Schneider, C. J. (2013). Divergence in coloration and ecological speciation in the *Anolis marmoratus* species complex. Molecular Ecology, 22(10), 2668–2682. 23611648 10.1111/mec.12295

[ece34650-bib-0051] Murcray, C. E. , Lewinger, J. P. , Conti, D. V. , Thomas, D. C. , & Gauderman, W. J. (2011). Sample size requirements to detect gene‐environment interactions in genome‐wide association studies. Genetic Epidemiology, 35(3), 201–210. 10.1002/gepi.20569 21308767 PMC3076801

[ece34650-bib-0052] Navas, C. A. (2002). Herpetological diversity along Andean elevational gradients: Links with physiological ecology and evolutionary physiology. Comparative Biochemistry and Physiology Part A: Molecular & Integrative Physiology, 133(3), 469–485. 10.1016/S1095-6433(02)00207-6 12443907

[ece34650-bib-0053] Prates, I. , Angilleta, M. J. Jr , Wilson, R. S. , Niehaus, A. C. , & Navas, C. A. (2013). Dehydration hardly slows hopping toads (*Rhinella granulosa*) from xeric and mesic environments. Physiological and Biochemical Zoology, 86(4), 451–457. 23799839 10.1086/671191

[ece34650-bib-0054] Prates, I. , Melo‐Sampaio, P. R. , de Oliveira Drummond, L. , Teixeira , M. , Rodrigues , … A. C. (2017). Biogeographic links between southern Atlantic Forest and western South America: Rediscovery, re‐description, and phylogenetic relationships of two rare montane anole lizards from Brazil. Molecular Phylogenetics and Evolution, 113, 49–58. 10.1016/j.ympev.2017.05.009 28502765

[ece34650-bib-0055] Prates, I. , & Navas, C. A. (2009). Cutaneous resistance to evaporative water loss in Brazilian *Rhinella* (Anura: Bufonidae) from contrasting environments. Copeia, 2009(3), 618–622. 10.1643/CP-08-128

[ece34650-bib-0056] Prates, I. , Rivera, D. , Rodrigues, M. T. , & Carnaval, A. C. (2016). A mid‐P leistocene rainforest corridor enabled synchronous invasions of the Atlantic Forest by Amazonian anole lizards. Molecular Ecology, 25(20), 5174–5186. 27564209 10.1111/mec.13821

[ece34650-bib-0057] Prates, I. , Rodrigues, M. T. , Melo‐Sampaio, P. R. , & Carnaval, A. C. (2015). Phylogenetic relationships of Amazonian anole lizards (*Dactyloa*): Taxonomic implications, new insights about phenotypic evolution and the timing of diversification. Molecular Phylogenetics and Evolution, 82, 258–268. 10.1016/j.ympev.2014.10.005 25451806

[ece34650-bib-0058] Prates, I. , Xue, A. T. , Brown, J. L. , Alvarado‐Serrano, D. F. , Rodrigues, M. T. , Hickerson, M. J. , & Carnaval, A. C. (2016). Inferring responses to climate dynamics from historical demography in neotropical forest lizards. Proceedings of the National Academy of Sciences of the United States of America, 113(29), 7978–7985. 27432951 10.1073/pnas.1601063113 PMC4961184

[ece34650-bib-0059] R Core Team (2018). R: A language and environment for statistical computing. Vienna, Austria: R Foundation for Statistical Computing Retrieved from https://www.R-project.org/

[ece34650-bib-0060] Raschperger, E. , Engstrom, U. , Pettersson, R. F. , & Fuxe, J. (2003). CLMP, a novel member of the CTX family and a new component of epithelial tight junctions. Journal of Biological Chemistry, 279, 796–804. 10.1074/jbc.M308249200 14573622

[ece34650-bib-0061] Rellstab, C. , Gugerli, F. , Eckert, A. J. , Hancock, A. M. , & Holderegger, R. (2015). A practical guide to environmental association analysis in landscape genomics. Molecular Ecology, 24(17), 4348–4370. 10.1111/mec.13322 26184487

[ece34650-bib-0062] Ribeiro‐Júnior, M. A. (2015). Catalogue of distribution of lizards (Reptilia: Squamata) from the Brazilian Amazonia. I. Dactyloidae, Hoplocercidae, Iguanidae, Leiosauridae, Polychrotidae, Tropiduridae. Zootaxa, 3983(1), 1–110. 26250019 10.11646/zootaxa.3983.1.1

[ece34650-bib-0063] Rodríguez, A. , Rusciano, T. , Hamilton, R. , Holmes, L. , Jordan, D. , & Wollenberg Valero, K. C. (2017). Genomic and phenotypic signatures of climate adaptation in an *Anolis* lizard. Ecology and Evolution, 7(16), 6390–6403. 28861242 10.1002/ece3.2985 PMC5574798

[ece34650-bib-0064] Sobral‐Souza, T. , Lima‐Ribeiro, M. S. , & Solferini, V. N. (2015). Biogeography of Neotropical Rainforests: Past connections between Amazon and Atlantic Forest detected by ecological niche modeling. Evolutionary Ecology, 29(5), 643–655. 10.1007/s10682-015-9780-9

[ece34650-bib-0065] Teixeira, M. , Prates, I. , Nisa, C. , Silva‐Martins, N. S. C. , Strüssmann, C. , & Rodrigues, M. T. (2016). Molecular data reveal spatial and temporal patterns of diversification and a cryptic new species of lowland *Stenocercus* Duméril & Bibron, 1837 (Squamata: Tropiduridae). Molecular Phylogenetics and Evolution, 94, 410–423. 26432394 10.1016/j.ympev.2015.09.010

[ece34650-bib-0066] Thomé, M. T. C. , Sequeira, F. , Brusquetti, F. , Carstens, B. , Haddad, C. F. , Rodrigues, M. T. , & Alexandrino, J. (2016). Recurrent connections between Amazon and Atlantic forests shaped diversity in Caatinga four‐eyed frogs. Journal of Biogeography, 43(5), 1045–1056. 10.1111/jbi.12685

[ece34650-bib-0067] Tollis, M. , Ausubel, G. , Ghimire, D. , & Boissinot, S. (2012). Multi‐locus phylogeographic and population genetic analysis of *Anolis carolinensis*: Historical demography of a genomic model species. PLoS ONE, 7(6), e38474 10.1371/journal.pone.0038474 22685573 PMC3369884

[ece34650-bib-0068] Van Damme, R. , Bauwens, D. , Castilla, A. M. , & Verheyen, R. F. (1989). Altitudinal variation of the thermal biology and running performance in the lizard *Podarcis tiliguerta* . Oecologia, 80(4), 516–524. 10.1007/BF00380076 28312838

[ece34650-bib-0069] Vitt, L. J. , Avila‐Pires, T. C. S. , Espósito, M. C. , Sartorius, S. S. , & Zani, P. A. (2003). Sharing Amazonian rain‐forest trees: Ecology of *Anolis punctatus* and *Anolis transversalis* (Squamata: Polychrotidae). Journal of Herpetology, 276–285.

[ece34650-bib-0070] Vitt, L. J. , Avila‐Pires, T. C. S. , Zani, P. A. , Sartorius, S. S. , & Espósito, M. C. (2003). Life above ground: Ecology of *Anolis fuscoauratus* in the Amazon rain forest, and comparisons with its nearest relatives. Canadian Journal of Zoology, 81(1), 142–156.

[ece34650-bib-0071] Vitt, L. J. , Cristina, T. , Avila‐Pires, S. , Zani, P. A. , & Espósito, M. C. (2002). Life in shade: The ecology of *Anolis trachyderma* (Squamata: Polychrotidae) in Amazonian Ecuador and Brazil, with comparisons to ecologically similar anoles. Copeia, 2002(2), 275–286. 10.1643/0045-8511(2002)002[0275:LISTEO]2.0.CO;2

[ece34650-bib-0072] Vitt, L. J. , Sartorius, S. S. , Avila‐Pires, T. C. S. , & Espósito, M. C. (2001). Life on the leaf litter: The ecology of *Anolis nitens tandai* in the Brazilian Amazon. Copeia, 2001(2), 401–412.

[ece34650-bib-0073] Yoder, J. B. , Stanton‐Geddes, J. , Zhou, P. , Briskine, R. , Young, N. D. , & Tiffin, P. (2014). Genomic signature of adaptation to climate in *Medicago truncatula* . Genetics, 196, 1263–1275. 24443444 10.1534/genetics.113.159319 PMC3982686

