# Supplementary material for: Local adaptation in mainland anole lizards: Integrating population history and genome–environment associations
Source: Ecol Evol. 2018 Nov 6;8(23):11932–44. doi: 10.1002/ece3.4650 (PMC6303772; doi:10.1002/ece3.4650)

Figure S1. Histograms of corrected p-values from genome-environment association analyses using LFMM.

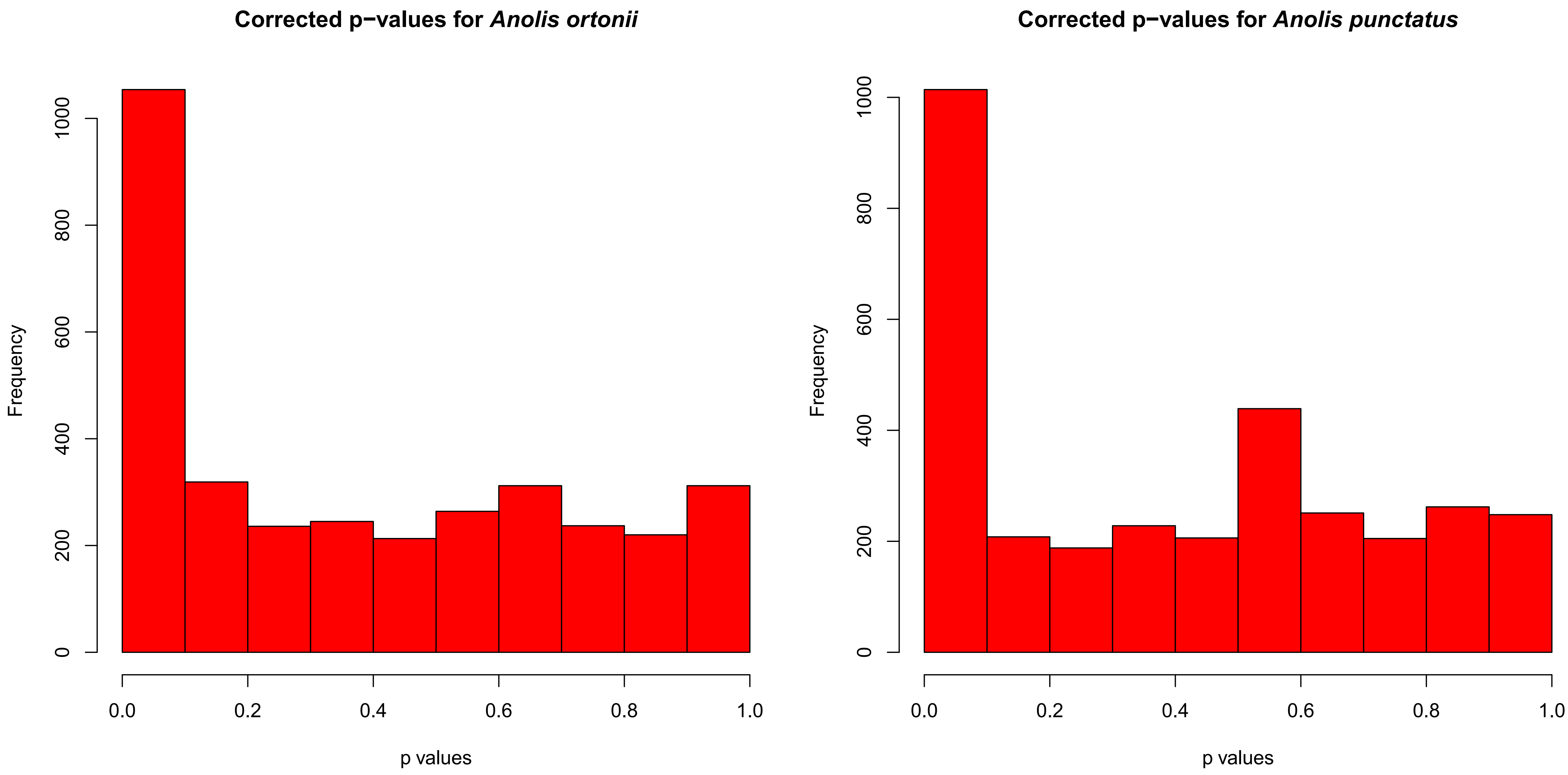

Supplement: Supplementary file 1 [file ECE3-8-11932-s001.pdf]
