# Supplementary material for: Local adaptation in mainland anole lizards: Integrating population history and genome–environment associations
Source: Ecol Evol. 2018 Nov 6;8(23):11932–44. doi: 10.1002/ece3.4650 (PMC6303772; doi:10.1002/ece3.4650)

Figure S2. Genetic PCA based on the entire SNP dataset (A) or on the candidate SNPs only (B) from *Anolis punctatus.* Red arrows indicate *A. punctatus* sample MTR 20798 from Pacaraima, a mid-elevation site (820 m above sea level) in the Guiana Shield region that overlaps climatically with Atlantic Forest sites (see Fig. 3).

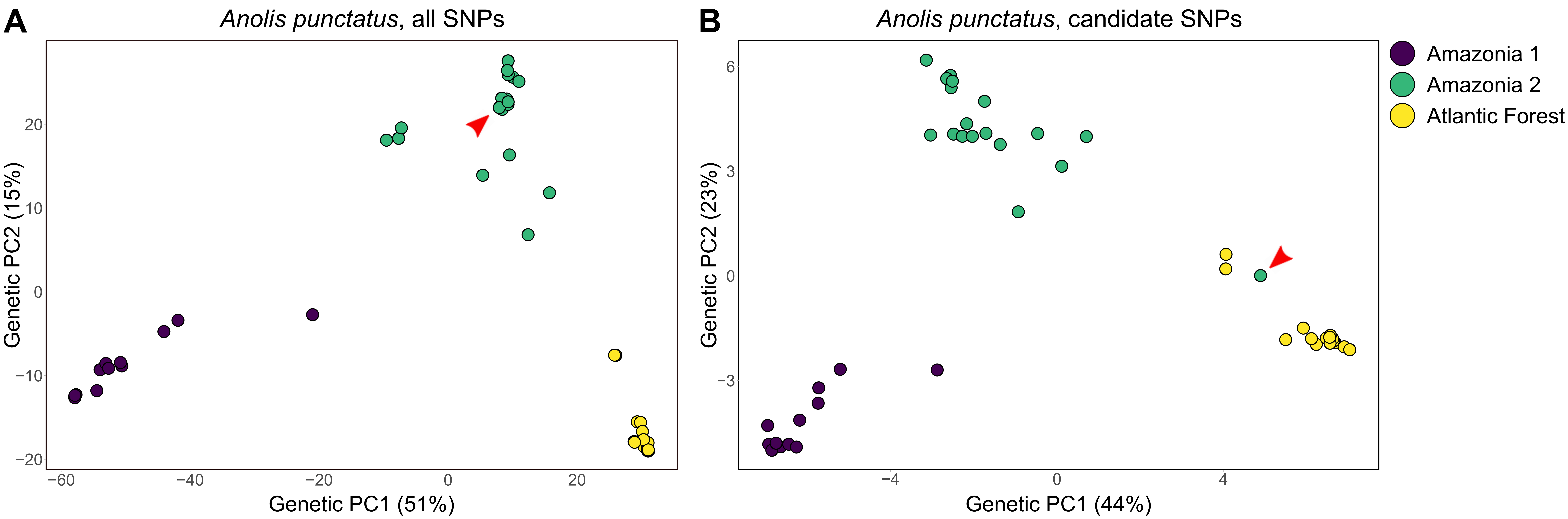

Supplement: Supplementary file 2 [file ECE3-8-11932-s002.pdf]
